# Editorial: 8th biennial international drug and alcohol research society conference 2022

**DOI:** 10.3389/adar.2024.13706

**Published:** 2025-02-14

**Authors:** Emmanuel S. Onaivi

**Affiliations:** Department of Biology, College of Science and Health, William Paterson University, Wayne, NJ, United States

**Keywords:** alcohol intoxication, nicotine, gut microbiome, epigenetics, neuroimmune interaction

Addiction to drugs and alcohol is an increasing substance use disorder (SUD), and public health problem worldwide, that is characterized by the compulsive use of addictive substances despite detrimental consequences for the individual and society. Globally, SUDs including alcohol use disorders (AUDs) affect more than 150 million people, and in the United States, AUDs alone afflict 29 million individuals, causing more than 140,000 deaths annually [1]. This is further complicated by the opioid crisis, which claims more than 100,000 lives every year [2]. The consequences of the COVID-19 pandemic, lockdown and isolation have resulted in excessive alcohol drinking behaviour, drug addiction, opioid overdose and death along with “Long Hauler” symptoms, comorbidity of neuroCOVID disorders, and now in transition to COVID-19 endemic status exacerbated SUDs. With treatment gaps and challenges on how SUDs are linked to dysbiosis, the implication that the gut-brain axis requires more understanding for comprehensive development of effective medications. Since bridging the SUD treatment gap and discovering new, more effective treatment medications are urgent priorities, there is a need for new research strategies and targets for the treatment of SUDs, as currently available therapies help only few that could benefit [2]. To address unmet needs in SUD treatment new frontiers in AI beyond CHATGPT with large quantitative AI, and combinations with advanced sensing may be useful to create new drugs for treating SUDs.

This Special Issue was put together to highlight the research advances and contributions made by some participants of the 2022, 8th biennial International Drug and Alcohol Research Society (IDARS) Conference in Nice, France. The goal of this Special Issue was to capture and present research data, reviews and discussions on the state of knowledge and the future of drug and alcohol addiction, which continues to be a global problem. Two research articles provided preclinical data using *in vivo* and *in vitro* techniques to evaluate the effects of alcohol, and four comprehensive review articles covered various molecular, gut microbiome, and neuroimmune effects of drugs and alcohol.

The research article by North et al. *, “Alcohol and pregnenolone interaction on cerebral arteries through targeting of vascular smooth muscle Ca* ^*2+*^ *- and voltage-gated K* ^*+*^ *channels of big conductance,”* investigated the effects of alcohol and pregnenolone (PREG) interaction on cerebral arteries in male and female C57BL/6J mice, to address the consequences in humans who might take pregnenolone supplements while binge drinking. The study showed that PREG at low concentrations synergized with alcohol on middle cerebral artery (MCA) constriction. However, this synergism was lost when both PREG and alcohol were studied at higher doses. Additional *in vitro* electrophysiological data acquisition and measurements of cerebral artery diameter provided evidence that PREG and alcohol converge on a common pathway to evoke cerebral artery constriction. Furthermore inhibition of Ca^2+^ and voltage-gated K^+^ large conductance (BK) channels by PREG and alcohol involves disruption of allosteric coupling to Ca^2+^ -driven gating. As PREG regulates several physiological processes, the study highlighted that a combination of PREG and alcohol may affect brain artery function. Of note is that AUDs occur in populations aged 65 and older, who may be at risk for cerebrovascular ischemic conditions.

The research article by Roberts et al., *“Alcohol induced behavioral and immune perturbations are attenuated by activation of CB2 cannabinoid receptors*,*”* investigated how CB2 cannabinoid receptors (CB2Rs) modulate the behavioral and neuroimmune perturbations using conditional knockout (cKO) mice with selective deletion of CB2Rs from dopamine neurons (DAT-*Cnr2*) and in a separate group of mice from microglia (Cx3Cr1-*Cnr2*). Motor function tests in activity monitors and wheel-running activity, rotarod performance, and alcohol preference tests were used to evaluate behavioral alterations induced by alcohol. An ELISA assay was used to determine the levels of pro-inflammatory cytokines, tumor necrosis factor-α (TNF-α), interleukin-6 (IL-6), interleukin-1α (IL-1α), and interleukin-1β (IL-1β) in the hippocampus of mice. The data showed that cell-type specific deletion of CB2Rs from dopamine neurons and microglia differentially altered the behavioral and alcohol preference tests and revealed that cell-type specific deletion of CB2Rs enhanced alcohol-induced inflammation. Pharmacological modification with the non-specific cannabinoid agonist WIN55212-2, reduced alcohol preference in the cell-type specific CB2R cKO and wild-type mice. The findings suggest that the involvement of CB2Rs in modulating behavioral and immune alterations induced by alcohol may be exploited as a potential therapeutic target in AUDs.

In their review, Crews et al., provided an overview of “Epigenetic regulation of microglia and neurons by proinflammatory signaling following adolescent intermittent ethanol (AIE) exposure and in human AUD.” A review of epigenetic mechanisms in response to neuroimmune signaling linked to high mobility group box 1 (HMGB1) plays a key cytokine-like molecule associated with brain proinflammatory signals in alcohol-induced changes. AIE-induced changes in neuroimmune gene expression in neurons, microglia and astrocytes increased adult drinking and preference, increasing anxiety and reward seeking. HMGB1 activates multiple proinflammatory receptors that spread proinflammatory receptors, including Toll-like receptors (TLRs) which mediate proinflammatory gene induction. Epigenetic mechanisms of AIE-induced AUD-like pathology have emerged as mechanisms of alcohol-induced changes in rodent and post-mortem human AUD hippocampus. HMGB1, neuroimmune signaling, epigenetic regulation of forebrain cholinergic neurons along with the hippocampal neurogenic niche provides a linkage between AIE and lifelong signaling associated with pathological behavior and hyperkatifeia that affect the development of AUD. Further studies are needed to develop therapeutic targets through anti-inflammatory and cell transcriptomes.

Next, two reviews also focused their attention on adolescent alcohol use. Hauser et al. presented *“Adolescent alcohol and nicotine exposure alters the adult response to alcohol use.”* Basic and clinical human research examining adolescent alcohol consumption and preclinical adolescent and adult alcohol consumption in rodents has revealed that adolescent alcohol and/or nicotine consumption/exposure can promote alcohol consumption during adulthood. The review summarized the knowledge on the effects of voluntary alcohol consumption during adolescence on models of adult alcohol consumption from humans to alcohol-preferring rat lines, including modeling of adolescent alcohol consumption and nicotine data. Mechanisms of the effects of alcohol and nicotine on dopamine and cholinergic systems are potential pharmacological targets and include varenicline, cholinesterase inhibitors, bupropion, lobeline and cytisine, which can reverse or prevent some of the deleterious changes during adulthood following adolescent alcohol consumption/exposure. Further research was suggested to identify and develop additional therapeutic targets for AUDs and co-use/abuse effects.

The review by Getachew et al., *“Adolescent alcohol drinking interaction with the gut microbiome: implications for adult alcohol use disorder”* discussed the growing importance of the bidirectional gut-brain axis as crucial for maintaining overall physiological homeostasis. The authors focused on the influence of adolescent alcohol use on the gut microbiota, as there is a high initiation of alcohol use in early adolescence that increases AUD in adulthood. Dysregulation during adolescent neurodevelopment including neuronal refinement and associated aberrant reward and impulsivity along with environmental and non-neuronal factors are contributing factors to neurodevelopmental impairments and AUD in adulthood. Furthermore, the roles of the gut microbiome and dysbiosis have been implicated in several peripheral and CNS diseases and AUDs. Mechanisms associated with gut microbiome-microglia interactions, including activation of Toll-like receptor signaling and inflammation-associated molecules suggest that bidirectional crosstalk between the gut and brain may influence fetal alcohol spectrum disorder (FASD). The bidirectional crosstalk between the gut and brain influences symptoms of FASD in individuals after birth in adolescent alcohol drinking and AUD. The gut microbiome, nutrients, and aspects of GPCR signaling are potential therapeutic targets in AUDs.

Vigorito and Chang contributed with *“Alcohol use and the pain system.”* They reviewed the mechanisms of nociception, nociceptive pain sensation, and pain perception on the contribution of the pain system to alcohol use, misuse, and dependence. The effects of alcohol at all levels of the pain system, such as neuroimmune interactions, molecular aspects of nociception, spinal, supraspinal, and affective-emotional circuits along with maladaptive homeostasis and allostasis, that contribute to the progression of AUDs were discussed and summarized.

In summary, this Special Issue consisting of two research articles and four review articles, provided pre-clinical research data and comprehensive review articles discussing multiple mechanisms associated with the effects of drugs and alcohol and highlighting challenges and treatment gaps for SUDs. Generative Artificial Intelligence (AI) systems have emerged as promising tools to improve individual health outcomes by streamlining diagnosis and treatment with clinical applications. While generative AI holds potential in the field of substance use disorders, caution is required as the functionalities of AI continue to evolve, as do the challenges of substance use disorders [3]. In addressing treatment gaps, AI beyond CHATGPT may offer useful opportunities to identify more urgent and effective treatment medications for SUDs.
